# Transfer freier Aminogruppen via α‐Aminierung von Carbonylen

**DOI:** 10.1002/ange.202304990

**Published:** 2023-06-05

**Authors:** Minghao Feng, Anthony J. Fernandes, Ana Sirvent, Eleonora Spinozzi, Saad Shaaban, Nuno Maulide

**Affiliations:** ^1^ Institut für Organische Chemie Universität Wien Währinger Straße 38 1090 Wien Österreich; ^2^ Christian-Doppler Labor für Entropieorientiertes Drug Design Josef-Holaubek-Platz 2 1090 Wien Österreich

**Keywords:** α-Aminocarbonyle, Peptide, Primäre Amine, Sulfonium [2,3]-Umlagerung

## Abstract

Eine Strategie zur direkten α‐Aminierung unfunktionalisierter Carbonylverbindungen wird berichtet. Unter Verwendung einer kommerziell verfügbaren Stickstoffquelle zur Übertragung der freien Aminogruppe (NH_2_) werden primäre α‐Aminocarbonylverbindungen unter besonders milden Bedingungen hergestellt. Die direkte Einführung einer ungeschützten, primären Aminogruppe ermöglicht in der Folge zahlreiche in situ Funktionalisierungen der erhaltenen Reaktionsprodukte, einschließlich Peptidkupplungen und Pictet–Spengler Cyclisierungen.

## Graphical abstract

Eine Strategie zum Aminogruppentransfer wurde entwickelt, die die Installation einer freien NH_2_‐Gruppe in α‐Position einer Carbonylfunktion unter milden Bedingungen ermöglicht. Die erhaltenen primären α‐aminierten Produkte können nachfolgend ohne Isolierung für in situ Peptidkupplungen oder Pictet–Spengler Cyclisierungen verwendet werden. Wir erwarten, dass die vorliegende Methode einen generellen Zugang zur präparativen Herstellung von Aminen eröffnet und neuartige Wege in der Synthese und medizinischen Studie von Peptidomimetics ermöglicht.

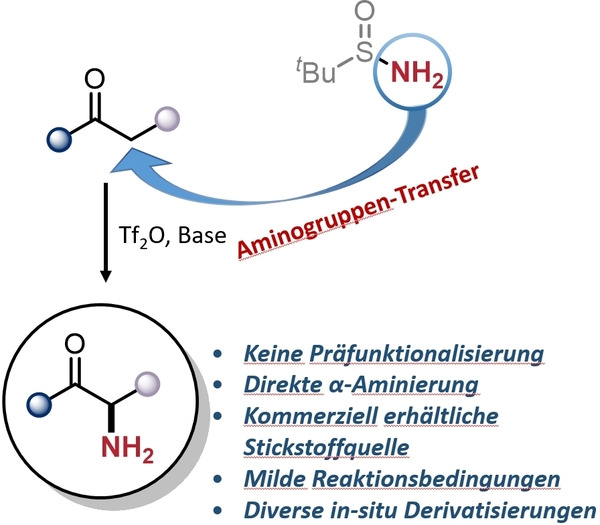

Stickstoff ist das vierthäufigste Element in pharmazeutisch aktiven Wirkstoffen (Schema 1A),[^1^, ^2^] wodurch die Entwicklung neuer Verfahren zur Installation von Aminogruppen an einer vorab genau definierten Position in einem Molekül von hoher Bedeutung ist.^[3]^ Die direkte Einführung einer freien, primären Aminogruppe (NH_2_) ist hierbei synthetisch besonders attraktiv, da so die Notwendigkeit zusätzlicher Transformationen zur deren Freisetzung umgangen wird. Bemerkenswerterweise sind in der Fachliteratur jedoch nur wenige Verfahren zur Herstellung primärer α‐Aminocarbonylverbindungen bekannt.^[4]^ Von besonderer Problematik sind hierbei die nicht zusammenpassenden elektronischen Eigenschaften des α‐Kohlenstoffatoms der Carbonylverbindung sowie des Aminostickstoffatoms, wodurch in vielen Fällen eine Präfunktionalisierung des Carbonylpartners in Form einer α‐Halogenierung notwendig wird (Schema 1b).^[5]^ Während entsprechende Aminierungen mit elektrophilen Aminierungsreagenzien eine wichtige Alternative darstellen,^[6]^ führen entsprechende Methoden typischerweise jedoch zunächst zu α‐Hydrazinyl‐ oder α‐Aminoxyprodukten, die mittels weiterer Folgereaktionen erst in die gewünschten primären Aminoderivate überführt werden müssen. 2019 berichteten Kürti und Mitarbeiter über eine einfache Synthese von primären α‐Aminoketonen ausgehend von Silylenolethern und *O*‐(2,4‐Dinitrophenyl)hydroxylamin bzw. Hydroxylamin‐*O*‐sulfonsäure (Schema 1B).^[4d]^ Methoden für eine direkte oxidative Aminierung, die die dargestellten Probleme umgeht, sind in der Literatur beschrieben, jedoch ist für diese die Verwendung von nukleophilen (meist cyclischen, sekundären) Aminen als Aminierungsreagenzien von entscheidender Bedeutung.^[7]^ Die direkte Installation einer primären NH_2_‐Gruppe in α‐Position einer Carbonylfunktion ist bisher nicht bekannt und verbleibt ein gleichermaßen herausforderndes wie attraktives Ziel.

**Scheme 1:**
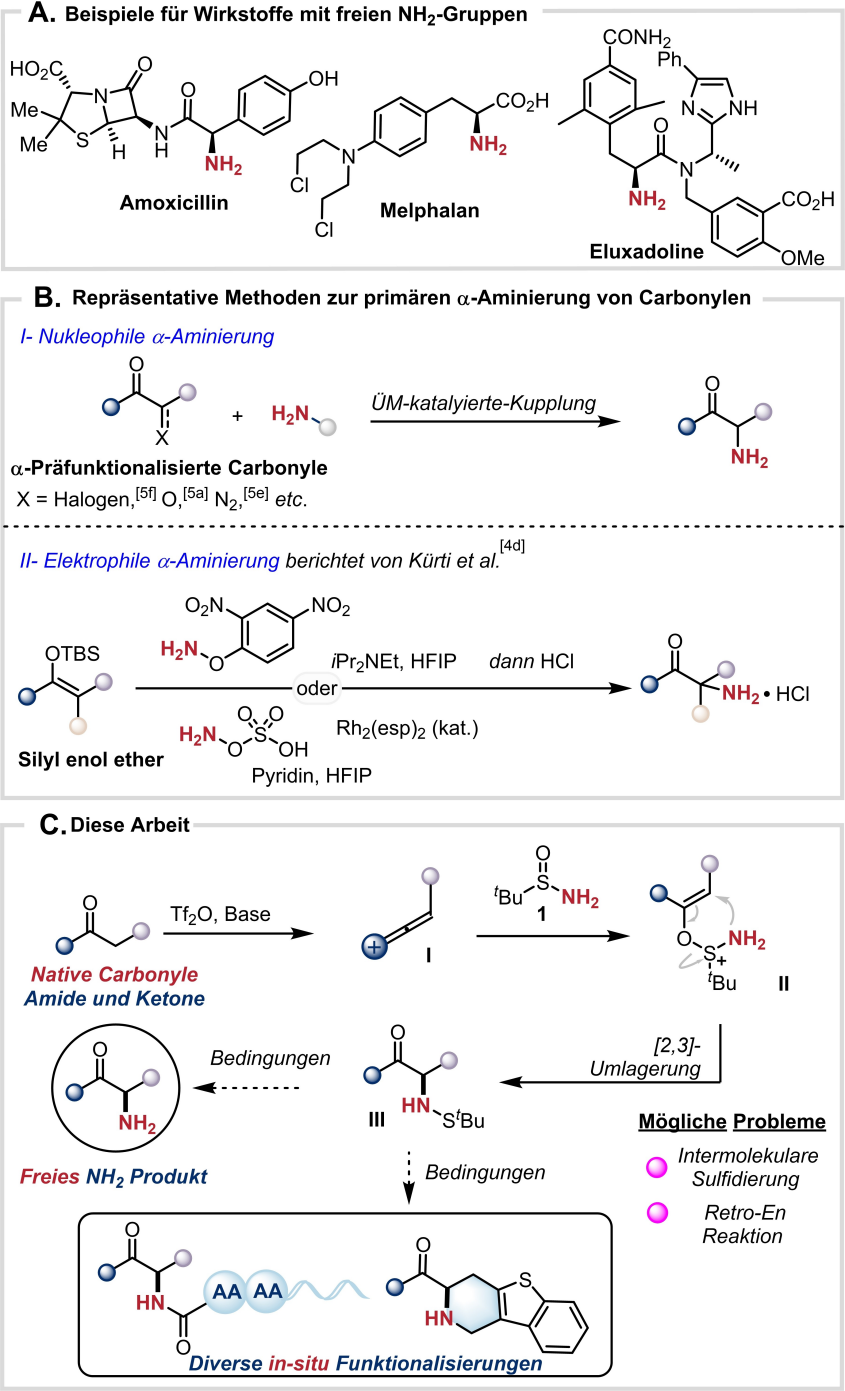
A) Beispiele für Wirkstoffe mit freien NH_2_‐Gruppen. B) Repräsentative Methoden zur primären α‐Aminierung von Carbonylen. C) Diese Arbeit: Freie α‐Aminierung unfunktionalisierter Amide und Ketone sowie verschiedene in situ Folgereaktionen.

Ladungsbeschleunigte Sulfoniumumlagerungen haben sich als nützliche Strategie für die Knüpfung von C−C und C−N Bindungen erwiesen.^[8]^ Kürzlich beschrieb unsere Gruppe erfolgreich eine direkte Kupplung von Amiden und Sulfiniminen, die die Synthese von β‐Aminoamiden via einer [3,3]‐sigmatropen Sulfoniumumlagerung ermöglicht.^[8i]^ Basierend auf diesen Ergebnisse stellten wir die Hypothese auf, dass die Umsetzung von *tert*‐Butylsulfinamid^[9]^ (**1**) mit einem geeigneten Vinylkation **(I)**[^10^, ^11^] in der Bildung einer neuen C−N Bindung resultieren und so einen direkten Zugang zur Einführung einer primären Aminogruppe in einem Schritt ermöglichen sollte. Als mögliche Unwägbarkeiten der beschriebenen Strategie wurden etwaige Nebenreaktionen von (**II**) wie z. B. intermolekulare Sulfidierung^[12a]^ bzw. Retro‐En‐Reaktion^[12b]^ evaluiert. Diese würden schlussendlich zu unerwünschten α‐Sulfanylcarbonylen bzw. den eingesetzten Startmaterialien führen. Abschließend müssten noch geeignete Bedingungen zur direkten Spaltung der N−S Bindung von Sulfenamid **III** identifiziert werden (Schema 1C).

Wir begannen unsere Studien zur α‐Aminierung von Amiden mit *N*,*N*‐Dimethyl‐4‐phenylbutanamid (**2 a**) als Modellsubstrat. Unter Verwendung von Bedingungen die sich in vorhergehenden Studien als optimal für elektrophile Amidaktivierung (Tf_2_O, 2‐Iodpyridin) erwiesen haben, führte die Addition von *tert*‐Butylsulfinamid **1** an das aus **2 a** resultierende Keteniminiumion nach anschließender saurer Aufarbeitung (HCl in Dioxan) zur Bildung des erwünschten α‐NH_2_ Amids **4 a** (68 % Ausbeute).^[8i]^ Zusätzlich konnten 15 % Startmaterial nach Aufarbeitung der Reaktionsmischung zurückgewonnen werden, deren Ursprung dabei vermutlich nicht in einem unvollständigen Umsatz von **2 a** liegt, sondern in der als Nebenreaktion ablaufenden Retro‐En‐Reaktion.^[12b]^ Im Gegensatz dazu konnten keinerlei, aus einer etwaigen α‐Sulfidierung resultierenden, Nebenprodukte beobachtet werden.^[12a]^

Im Folgenden sollte die breite Anwendbarkeit unserer Methode demonstriert werden. Zu diesem Zweck wurden zahlreiche Amide unter den beschriebenen Bedingungen umgesetzt und lieferten die gewünschten primären Amine in guten Ausbeuten (**4 b**–**h**, Schema 2). Von besonderer Bedeutung sind hierbei die morpholin‐ (**4 d**), weinrebamid‐ (**4 e**), und indolinylbasierten (**4 f**) Produkte, die für Folgereaktionen zur Synthese anderer Carbonylverbindungen geeignet sind. Die Anwesenheit eines Trifluoromethylsubstituenten nahe dem reaktiven Zentrum hatte keinen negativen Einfluss auf das Reaktionsergebnis (**4 h**). Die entwickelte Methode toleriert eine Vielzahl an funktionellen Gruppen einschließlich Alkene (**4 i, 4 j**), Alkine (**4 k**), Allylether (**4 l**), Ester (**4 n**), Nitrile (**4 o**) und Phthalimide (**4 p**). Überraschenderweise, wurde für ein Amid mit einer Ketofunktion eine vergleichsweise geringe isolierte Ausbeute (38 %) für das angestrebte aminierte Produkt **4 m** erhalten. Als mögliche Ursache hierfür könnten unerwünschte Kondensierungsreaktionen in Frage kommen, die während der sauren Aufarbeitung zur Spaltung der N−S Bindung ablaufen. Verschiedene Heteroaromaten, sowie substituierte Aromaten wurden ebenso toleriert (**4 q**–**t**) und ein cyclopropylsubstituiertes Startmaterial **3 u** konnte ebenso erfolgreich in das korrespondierende Produkt (**4 u**) überführt werden. Etwaige Nebenreaktionen, die aus einer Ringöffnung oder einer Zersetzung des Startmaterials resultieren, wurden nicht beobachtet. Bemerkenswerterweise konnte ein dreizehngliedriges Lactam in guter Ausbeute in das entsprechende α‐Aminoderivat (**4 v**) überführt werden.

**Scheme 2:**
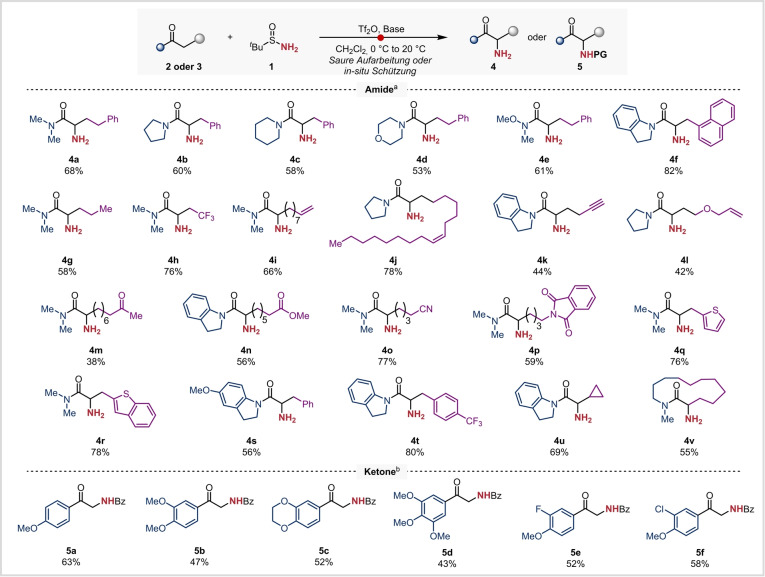
Bedingungen a) Aminierung von Amiden: Reaktionen wurden im 0.2 mmol Maßstab durchgeführt: Amid (1.0 Äquiv.), 2‐Iodopyridin (2.2 Äquiv.), Tf_2_O (1.2 Äquiv.), *tert‐*Butylsulfinamid (2.0 Äquiv.), CH_2_Cl_2_ (0.1 M), 0 °C nach 20 °C, 24 h, dann saure Aufarbeitung (HCl in Dioxan, 20 °C, 16 h). Bedingungen b) Aminierung von Ketonen: Reaktionen wurden im 0.2 mmol Maßstab durchgeführt: Keton (1.0 Äquiv.), DTBMP (1.2 Äquiv.), Tf_2_O (1.2 Äquiv.), *tert*‐Butylsulfinamid (2.0 Äquiv.), CH_2_Cl_2_ (0.1 M), 0 °C nach 20 °C, 24 h dann Schutzgruppenaufarbeitung (BzCl, NEt_3_, CH_2_Cl_2_, 20 °C, 16 h). DTBMP: 2,6‐Di‐*tert*‐butyl‐4‐methyl‐pyridin. Siehe Hintergrundinformationen für Details.

Elektronenreiche Arylketone stellen ebenso geeignete Substrate für eine elektrophile Aktivierung dar,^[11]^ was als Grundvoraussetzung für die vorliegende α‐Aminierungsstrategie betrachten werden kann. Hierbei resultierte die saure Aufarbeitung der Reaktionsmischung jedoch in Nebenreaktionen wie z. B. der Kondensation der generierten primären Aminofunktion mit der Ketogruppe des Startmaterials. Um dieses Problem zu umgehen wurde eine in situ Benzoylierung der freien NH_2_‐Gruppe durchgeführt, wodurch die Isolierung der korrespondierenden Reaktionsprodukte in Form der N‐geschützten Derivate ermöglicht wurde (**5 a**–**f**).

Die in dieser Arbeit dargestellte Reaktion sticht dadurch heraus, dass sie eine der wenigen Transformationen ist, die die direkte Installation einer freien NH_2_ Gruppe ermöglicht. Ein weiterer Vorteil der entwickelten Methode ist die Möglichkeit weiteren Nutzen aus der vielfältigen Chemie der erhaltenen Amine mittels anschließender in situ Funktionalisierungen zu schlagen. So sind freie NH_2_‐Gruppen beispielsweise für Peptidkupplungen geeignet und Amine **4 d**, **4 v** und **4 q** konnten erfolgreich in situ in einem Ein‐Topf‐Verfahren mit verschiedenen Aminosäuren gekuppelt werden. Es sollte an dieser Stelle erwähnt werden, dass dies einer formalen und bis dato nicht beschriebenen “α‐Peptidierung” von zuvor unfunktionalisierten Amiden in einem einzigen Schritt gleichkommt, wobei die entsprechenden Peptide **6 a**–**d** in exzellenten Ausbeuten erhalten werden (Schema 3A). Interessanterweise wurde Verbindung **6 d** in sehr guter Ausbeute als 9 : 1 Mischung von Diastereomeren erhalten, wenn enantiomerenreines Sulfonamid **(*R*)‐1** verwendet wurde.

**Scheme 3:**
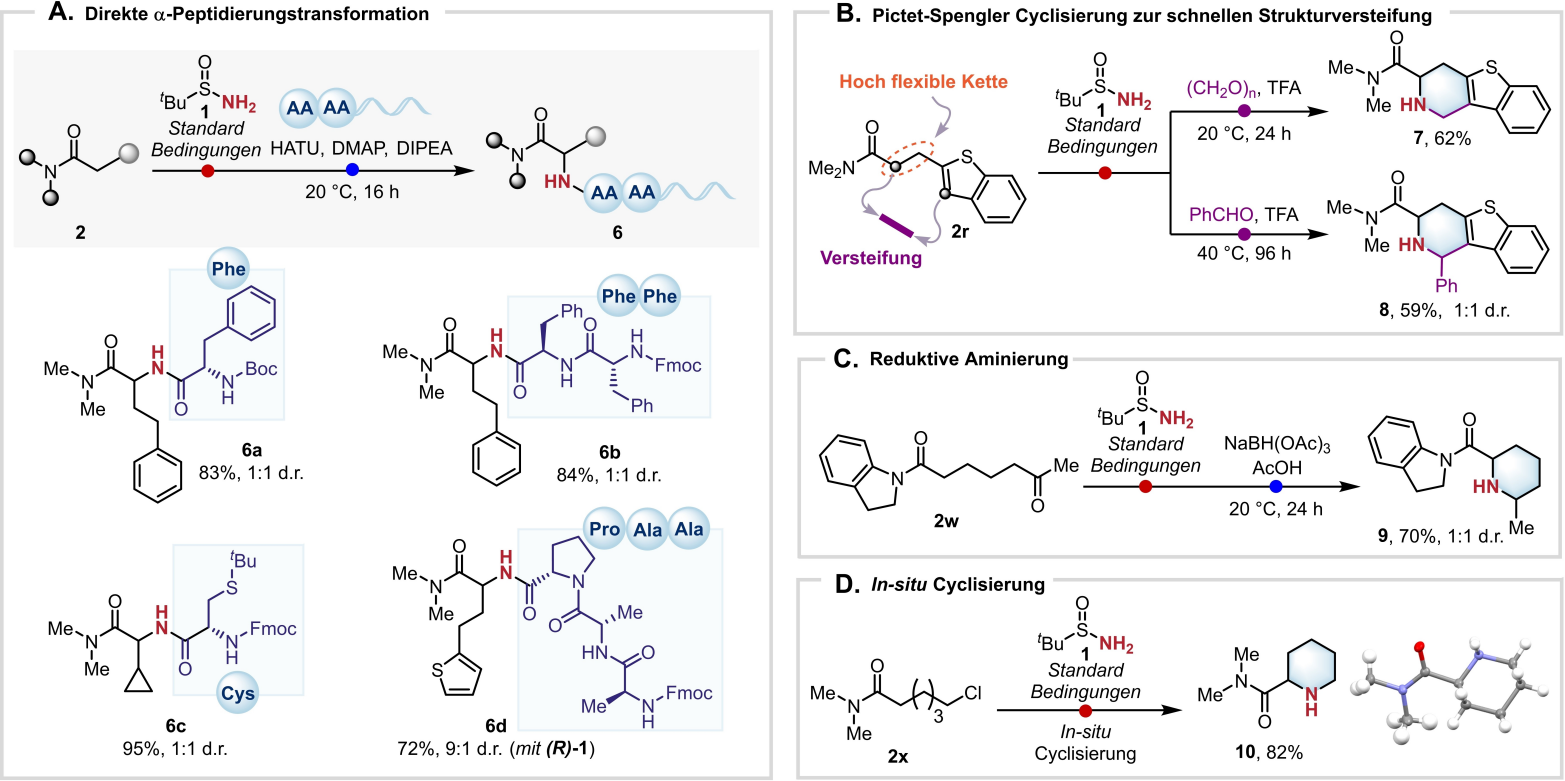
α‐Aminierung/Funktionalisierung von Amiden. Reaktionen wurden im 0.2 mmol Maßstab durchgeführt. A) Direkte α‐Peptidierung: Amid (1.0 Äquiv.), 2‐Iodpyridin (2.2 Äquiv.), Tf_2_O (1.2 Äquiv.), *tert*‐Butylsulfinamid (2.0 Äquiv.), CH_2_Cl_2_ (0.1 M), 0 °C nach 20 °C, 24 h dann Aminosäure (2.0 Äquiv.), HATU (2.2 Äquiv.), DIPEA (2.2 Äquiv.), CH_2_Cl_2_ (0.04 M), 20 °C, 3 h. B) α‐Aminierung/Pictet–Spengler: Amid (1.0 Äquiv.), 2‐Iodpyridin (2.2 Äquiv.), Tf_2_O (1.2 Äquiv.), *tert*‐Butylsulfinamid (2.0 Äquiv.), CH_2_Cl_2_ (0.1 M), 0 °C nach 20 °C, 24 dann Aldehyd (2.0 Äquiv.), TFA (4.0 Äquiv.), 20 °C, 24 h oder 40 °C, 96 h. C) α‐Aminierung/Reduktive Aminierung: Amid (1.0 Äquiv.), 2‐Iodpyridin (2.2 Äquiv.), Tf_2_O (1.2 Äquiv.), *tert*‐Butylsulfinamid (2.0 Äquiv.), CH_2_Cl_2_ (0.1 M), 0 °C nach 20 °C, 24 h dann NaBH(OAc)_3_ (2.0 Äquiv.), AcOH (1.0 Äquiv.), 20 °C, 24 h. D) In situ Cyclisierung: Amid (1.0 Äquiv.), 2‐Iodpyridin (2.2 Äquiv.), Tf_2_O (1.2 Äquiv.), *tert*‐Butylsulfinamid (2.0 Äquiv.), CH_2_Cl_2_ (0.1 M), 0 °C nach 20 °C, 24 h, dann 4 M HCl in Dioxan, 20 °C, 16 h. HATU: Azabenzotriazol‐tetramethyluronium‐hexafluorophosphat. DMAP: 4‐Dimethylaminopyridin. DIPEA: *N*,*N*‐Diisopropylethylamin. TFA: Trifluoressigsäure.

Darüber hinaus konnte gezeigt werden, dass primäre Amine wie **4 r** geeignete Substrate für Pictet–Spengler Cyclisierungen darstellen. In Abhängigkeit des Reaktionspartners (Paraformaldehyd oder Benzaldehyd) wurden hierbei die Piperidine **7** und **8** erhalten (Schema 3B).^[13]^ Diese Transformation erlaubt die Verknüpfung des α‐Kohlenstoffatoms der Amidgruppe in **2 r** mit der 3‐position des Heteroaromaten, wobei die eingeführte Aminogruppe quasi als “Stütze” agiert und so die gesamte Struktur versteift – eine potentiell hochgradig nützliche Transformation in der Wirkstoffentwicklung. Ketoamid **2 w** wurde nachfolgend mittels einer Eintopf α‐Aminierung/reduktive Aminierungssequenz in Piperidin **9** überführt (Schema 3C).^[14]^ Abschließend konnte ω‐chlorsubstituiertes Amid **2 x** mittels einer tandem α‐Aminierung/Cyclisierung in sehr guter Ausbeute in Piperidin **10** überführt werden (Schema 3D).^[15]^

Aus mechanistischer Sicht gehen wir davon aus, dass zunächst ein nukleophiler Angriff des *tert*‐Butylsulfinamids auf die Keteniminiumspezies **I** oder deren Analogon (**I′**) erfolgt (Schema 4). Die so gebildete Sulfoniumspezies **II** durchläuft dann eine ladungsbeschleunigte [2,3]‐Umlagerung,[^8j^, ^16^] wobei Sulfenamid **III** gebildet wird.^[17]^ Bemerkenswerterweise konnten sowohl Sulfenamide vom Typ **III**, als auch die primären α‐aminierten Produkte vor der abschließenden sauren Aufarbeitung mittels LC/MS detektiert werden. Die Aufarbeitung vervollständigt die angestrebte N−S Bindungsspaltung somit lediglich.

**Scheme 4:**
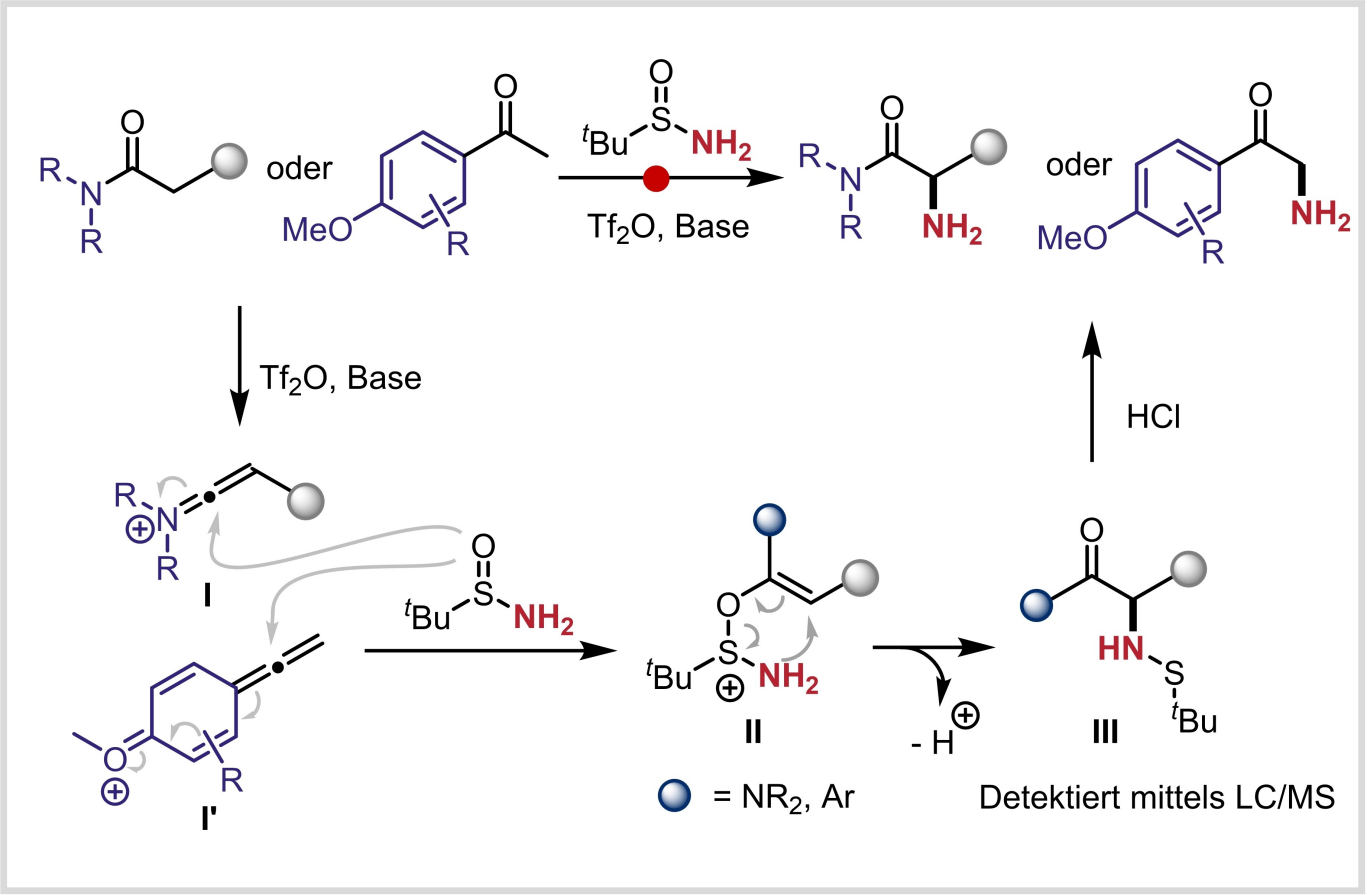
Möglicher Mechanismus für die direkte α‐Aminierung von Ketonen und Amiden.

Zusammenfassend lässt sich festhalten, dass eine einfache Strategie zur direkten Einführung von primären Aminogruppen α zu Carbonylverbindungen unter milden Bedingungen entwickelt wurde. Die vorliegende Methode erlaubt eine Vielzahl an nachgeschalteten in situ Derivatisierungen, einschließlich Peptidkupplungen und Pictet–Spengler Cyclisierungen. Wir gehen davon aus, dass diese Methode einen allgemeinen Zugang zur Synthese von unnatürlichen α‐Aminosäurederivaten bietet und in Zukunft neuartige Wege für die Herstellung von Peptidomimetica sowie deren medizinischer Erforschung eröffnet.

## Interessenkonflikt

Die Autoren erklären, dass keine Interessenkonflikte vorliegen.

## Supporting information

As a service to our authors and readers, this journal provides supporting information supplied by the authors. Such materials are peer reviewed and may be re‐organized for online delivery, but are not copy‐edited or typeset. Technical support issues arising from supporting information (other than missing files) should be addressed to the authors.

Supporting Information

## Data Availability

Die Daten, die die Ergebnisse dieser Studie unterstützen, sind in den Hintergrundinformationen zu diesem Artikel verfügbar.
